# The breakdown of social camouflagin: midlife emergence of autistic traits in women

**DOI:** 10.1192/j.eurpsy.2026.11538

**Published:** 2026-07-31

**Authors:** L. M. D.-P. Barros, R. D. A. Gómez, P. S. Aranda, I. R. Blanco, C. M. Lorenzo, A. S. Chillón, R. A. S. Limo

**Affiliations:** SACYL, Salamanca, Spain

## Abstract

**Introduction:**

High fuinctioning autism spectrum disorder (ASD) in women frequently goes unrecognized, with many receiving diagnosis after years of social camouflaging and misattributed symptoms. Midlife introduces new challenges that can unmask long compensated autistic traits. This review aims to explore whether midlife and aging act as critical periods that unmask high functioning ASD in women. We hypothesize that hormonal shifts, compounded by life stressors, exacerbate underlying autistic traits that lead to new clinical presentations.

**Objectives:**

This review aims to explore whether midlife and aging act as critical periods that unmask high functioning ASD in women. We hypothesize that hormonal shifts, compounded by life stressors, exacerbate underlying autiostic traits that lead to new clinical presentations.

**Methods:**

A narrative literature review by searching MEDLINE (vía PubMed), EMBASE, PsychINFO, and Cochrane Library for articles published from 2010 to 2025. We used controlled vocabulary and key words including MeSH terms (“Autistic Disorder”, “Asperger Syndrome”, “Women”, “Menopause”, “Aging”) and EMtree terms (“autistic sprectrum disorder”, “female”, “midlife crisis!, “camouflaging behaviour”. We combined these terms with Boolean operators AND and OR. We prioritsed studies published in English or Spanish, involving adult women with high functionin ASD and addressing diagnostic features, psychosocial outmcomes, or health impacts during midlife.

**Results:**

Findings indicate that many autistic women enter midlife with profound fatigue from years of masking, high susceptibility to anxiety and depression. Menopausal changes and shrinking social networks further exacerbate isolation. While diagnosis at this stage often provides explanatory value and guides supportive interventions, it also raises questions about the thesholds for labeling lifelong neurodevelopmental patterns in the absence of functioning impairment.

- Bargiela S et al.The expriences of late- diagnosed women with autism spectrum conditions: An investigation of the female autism phenotype. J Autism Dev Disord. 2016; 46 (19): 3281-94
- Lai MC et al. Sex/gender differences and autism: setting the scne for future research. J Am Acad Child Adolec Psychiatry. 2015; 54(1(: 11-24
- Hull L, et al. Autism spectrum disorder ad social camouflaging. Austism. 2017; 21(&): 702-717.
- Moseley RL, et al. Brain and hormonal changes in autistic women. J Neurodev Disord. 2021; 13(1):9.

**Conclusions:**

Recognizing ASD in women during midlife requires a refined, gender-sensitive approach. The decision to pursue or communicate a formal diagnosis should weight the potential for empowerment and targeted psychoeducation against the risks of unnecessary medicalization. Future research should refine diagnostic criteria and develop specific supports that acknowledge the unique trajectories of autistic women across the lifespan.

**Disclosure of Interest:**

None Declared

